# Diversity of Root System Architecture in Mediterranean Maize Inbred Lines Provides New Breeding Opportunities to Improve Stress Resilience and Resource Efficiency

**DOI:** 10.3390/plants15060935

**Published:** 2026-03-18

**Authors:** Rongli Shi, Dominic Knoch, Ana López-Malvar, Narendra Narisetti, Evgeny Gladilin, Thomas Altmann

**Affiliations:** 1 Leibniz Institute of Plant Genetics and Crop Plant Research (IPK), OT Gatersleben, 06466 Seeland, Germany; shi@ipk-gatersleben.de (R.S.); knochd@ipk-gatersleben.de (D.K.); alopezmalvar@uvigo.gal (A.L.-M.); narisetti@ipk-gatersleben.de (N.N.);; 2 Biología Vegetal y Ciencias del Suelo, Agrobiología Ambiental, Calidad de Suelos Y Plantas (UVIGO), Unidad Asociada a La MBG (CSIC), Universidad de Vigo, As Lagoas Marcosende, 36310 Vigo, Spain

**Keywords:** high-throughput phenotyping, maize (*Zea mays*), diversity, rhizotron, root system architecture (RSA)

## Abstract

A detailed characterization of root system architecture (RSA) and growth dynamics is key to develop stress-resilient maize varieties. We evaluated sixty-five Mediterranean maize inbred lines using automated high-throughput phenotyping under controlled conditions. Shoot and root traits were extracted from imaging data during early vegetative development, revealing significant genotype-specific variation in root biomass-related traits (total root length, total root volume), root architecture (root angle, root system depth, root system width), and relative growth rates. Notably, lines previously classified as heat and drought stress-resilient or stress-sensitive based on above-ground development did not group according to particular root traits, indicating that multiple strategies may underlie tolerance to combined stress. We identified lines with contrasting RSA, including deeper roots, shallower roots, or overall larger root systems, that offer new opportunities for resilience breeding. Our results underscore root traits as critical yet underexploited targets for improving stress resilience and resource efficiency.

## 1. Introduction

Maize (*Zea mays*), one of the most important crops worldwide, plays a vital role in global food security. It is grown across the globe and can be processed into a variety of products, including food, animal feed, industrial goods, and biofuel [1,2]. However, maize production is increasingly challenged by various abiotic stresses, particularly those associated with adverse climatic scenarios. Current climate conditions, such as prolonged droughts, flooding episodes, irregular precipitation patterns, and extreme heat waves, negatively affect crop production worldwide. Such adverse events will occur more frequently in future decades as predicted by The Intergovernmental Panel on Climate Change (IPCC) [3]. Drought and heat stress can disrupt key physiological processes, leading to changes at the morphological, physiological, biochemical, and molecular levels, ultimately reducing plant growth and yield [4,5]. It is reported that global maize yields are reduced by about 39% due to drought stress [6]. Therefore, it is crucial to develop climate-smart maize genotypes to enhance environmental stress tolerance and boost maize production.

Roots play pivotal roles in anchorage and the acquisition of soil-based resources for plants [7,8]. In addition, roots contribute significantly to plant adaptation to stressful conditions. Previous research showed that there is natural diversity in root system architecture (RSA) in many plant species, such as Arabidopsis [9], wheat [10], or sorghum [11]. Maize, likewise, has been reported to exhibit genotypic variation in RSA under favourable growing conditions, as well as during stress [12,13,14,15]. Furthermore, phenotypic diversity can enhance a plant’s ability to respond to various environmental stresses, such as phosphorus deficiency or drought [16,17]. Under drought stress, total root length and root dry weight were reported to be decreased in plants [18], particularly in drought sensitive cultivars [19]. As one strategy to cope with drought, the root system can increase the fibrous root number, minimize the lateral root diameter, and form more root cortical aerenchyma [8,20]. Root angle is considered to be an important drought-adaptive trait and has been demonstrated to be positively correlated with shoot biomass and grain yield under drought conditions [21,22]. Steep root systems (small root angle) enable deeper rooting and to retrieve water and mobile nutrients in deep soil layers [23,24]. Many researchers have suggested that dissecting root system variations could enhance crop yield and sustainability under drought conditions. Therefore, a highly detailed investigation of RSA in climate-adapted lines is crucial to harness existing diversity and to accelerate the development of stress-resilient varieties and advancing agronomic improvement.

The development of maize varieties with a more advantageous root system holds great potential for improving water and nutrient use efficiency. In recent years, image-based root phenotyping has gained significant popularity due to technical advances [12,25]. Among various root phenotyping platforms, systems such as rhizoboxes or rhizotrons provide a standardized growth environment. Integrating such systems with digital non-invasive high-throughput methods has been widely adopted in the past few years [15,26]. This has allowed the reliable and precise assessment of root phenotypes and growth behaviour. Advances in image processing applications have enabled high-throughput phenotyping of roots and contributed significantly to our understanding of root function and its genetic basis [27]. This has enabled the identification of many candidate genes associated with root architecture in maize through genome-wide association studies (GWAS) [28,29], some of which were further validated [30,31]. Hence, exploring root phenotypes is essential to uncover the complex genetic mechanisms underlying maize root development and to support related breeding efforts. Additionally, simultaneously evaluating plant shoots and roots provides whole-plant-level insights by revealing the coordinated communication and physiological interdependence between these two systems, which is crucial for optimizing growth and resource use efficiency [32]. In our previous work, the morphological and physiological responses of 106 Mediterranean maize inbred lines to combined drought and high temperature were assessed [33]. These lines could potentially serve as genetic resources to mine alleles to enhance the tolerance to drought and/or high temperature stresses. They were classified as stress-resilient, stress-sensitive or intermediate based on a stress adaptability index. Following these evaluations [33], 65 inbred lines evaluated in our previous work were selected, and their constitutive RSA was assessed in a rhizotron-based high-throughput phenotyping facility in the present study. A well-developed root system is generally considered an important foundation for stress adaptation in plants [34]. We hypothesized that well adapted, stress-resilient lines should develop a larger and more efficient RSA already under non-stress growth conditions, enabling them to better withstand upcoming stress periods. Therefore, the objectives of this study were to (1) characterize the RSA of lines differing in their tolerance to combined heat and drought stress, (2) test whether plants exhibit preadaptation to adverse environmental conditions or employ multiple strategies in response to stress, and (3) identify promising lines for breeding programmes aimed at improving root systems, thereby enhancing heat and drought tolerance in maize.

## 2. Results

### 2.1. Mediterranean Maize Lines Displayed High Variation in Shoot Biomass

**Figure 1:**
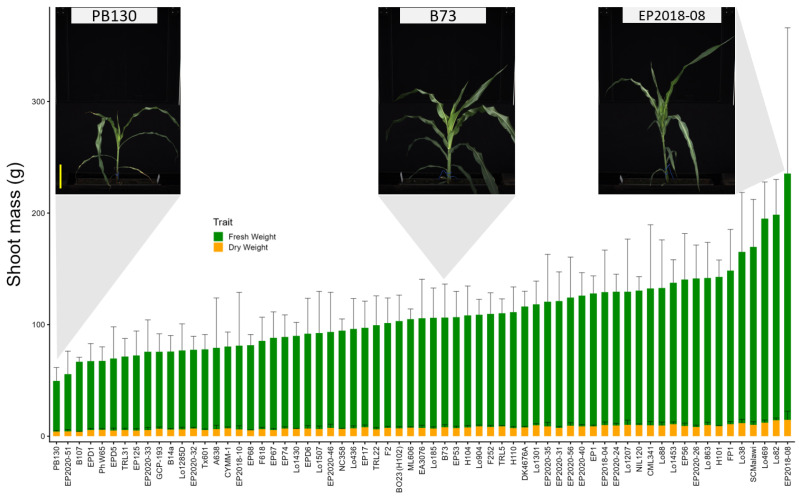
Natural variation in shoot biomass among 65 Mediterranean maize lines grown in the rhizotron system. Fresh weight (green) and dry weight (yellow) were determined at harvest, 27 days after sowing (DAS). The bars denote mean of four biological replicates and standard deviation for each line are indicated by error bars. Shoot images from three representative lines (PB130, B73, EP2018-08) are presented. The yellow scale bar represents 15 cm.

At harvest, 27 days after sowing (DAS), a wide variation in shoot biomass was observed among the selected 65 maize lines (Figure 1). Detailed information for these lines is provided in Table S1. Line EP2018-08 showed the highest shoot fresh weight (FW) and dry weight (DW), although with high variation among replicates, values were 4.7 times higher than those of PB130 (lowest FW) and 3.7 times higher than those of B107 (lowest DW), respectively. Significant phenotypic variation between genotypes was observed for both traits (ANOVA *p* < 0.001).

### 2.2. Manual Measurements Showed Significant Correlations with Specific Image-Derived Traits

**Figure 2:**
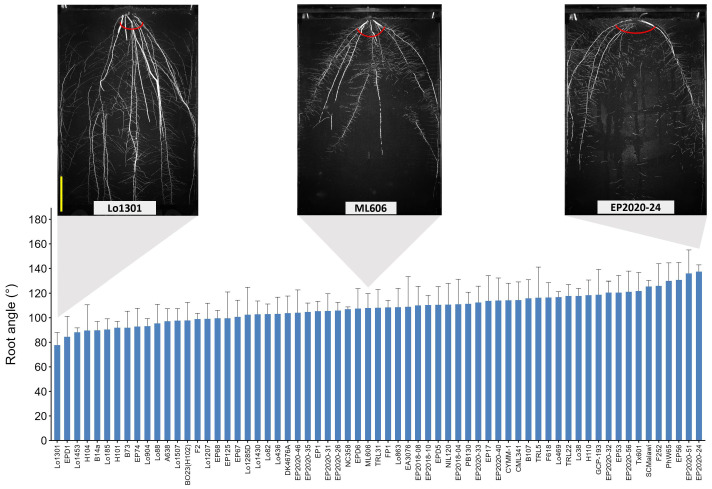
Root angle (°) of the tested 65 maize lines measured manually from Image J at 27 days after sowing (DAS). The bars denote the mean of four biological replicates, and standard deviations for each line are displayed. Root images from three representative lines (Lo1301, ML606, EP2020-24) are presented, with their root angles indicated in red. The yellow scale bar corresponds to 15 cm.

Phenotypic traits were extracted from the daily images, including four shoot traits obtained using DeepShoot software (https://ag-ba.ipk-gatersleben.de/ds.html (accessed on 15 March 2026)) [35] and 48 root traits calculated by the fully automated Root Image Analysis software (faRIA) (https://ag-ba.ipk-gatersleben.de/faria.html (accessed on 15 March 2026)) [36]. However, the ‘root angle’ trait, defined as the angle at which a root grows in relation to gravity or other stimuli [37] could not be extracted directly through automated image analysis. Therefore, this parameter (RA_m) was manually determined from the images taken on the final day (27 DAS), using Image J software (https://imagej.net/ij/ (accessed on 15 March 2026)). The average root angle across all lines was 108°, with values ranging from 78° to 137°. Lines Lo1301, EPD1, Lo1453 had the narrowest root angles, while EP2020-24, EP2020-51 and EP56 had the widest root angles (Figure 2). Additionally, we calculated the ratio of root system depth (geometrical_y_p99_wo) to root system width (geometrical_x_dq95), denoted as dep_wid_r, to assess whether it could serve as a proxy for root angle using image-derived traits.

**Figure 3:**
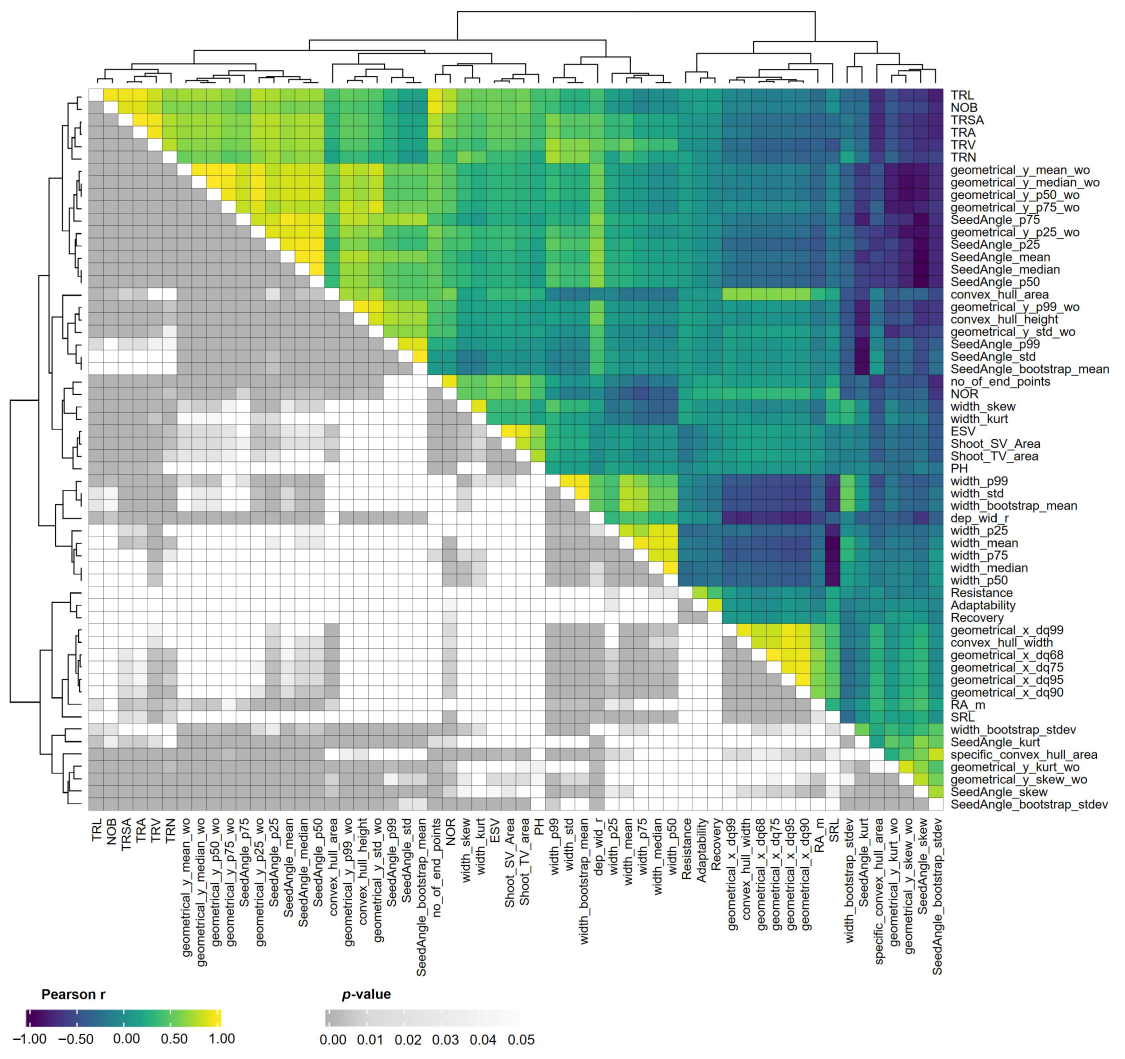
Correlation analysis of shoot and root architectural traits measured in 65 maize lines at 27 days after sowing. The two colour scales represent the Pearson correlation coefficient (r; viridis colour scale), and the *p*-value (grey colour scale), respectively. PH: plant height; ESV: estimated shoot volume; TRA: total root area; TRL: total root length; TRV: total root volume; TRSA: total root surface area; SRL: specific root length; NOR: number of regions; NOB: number of branching points; dep_wid_r: the ratio of root system depth to width. The abbreviations for all trait names are given in Supplementary Information File S1.

To examine correlations between manually measured and image-derived traits, as well as among image-derived traits themselves, we analyzed all variables, including manually measured RA_m, total root number (TRN), and three stress indices from our previous study [33] (Figure 3). Shoot biomass-related traits, including estimated shoot volume (ESV), shoot area from the side view (Shoot_SV_area), shoot area from the top view (Shoot_TV_area) and the shoot architecture-related trait, plant height (PH) grouped together. Similarly, root biomass-related traits, total root length (TRL), total root surface area (TRSA), total root area (TRA), total root volume (TRV) and TRN clustered together. These traits were found to be highly and significantly correlated with each other. PH and ESV both showed positive correlations with TRL, TRA, TRSA and TRV. The Pearson correlation coefficients between ESV and TRL, TRA, and TRV were r = 0.56 ***, r = 0.55 ***, and r = 0.49 ***, respectively. The orientation-related parameters whose names contained ‘SeedAngle’ were distributed across multiple clusters and did not show a clear pattern of correlation with other traits. RA_m showed a positive correlation with root system width (r = 0.62 ***) and related traits, while it was negatively correlated with the root system depth-related parameter, geometrical_y_p25_wo (r = −0.45 ***) and the root system depth to width ratio (dep_wid_r; r = −0.58 ***). Manually counted total root number (TRN) positively correlated with TRV (r = 0.78 ***) and number of branching points (NOB; r = 0.62 ***). Interestingly, the stress resistance index showed a significant negative correlation with root diameter (width_mean; r = −0.29 *; Figure 3).

### 2.3. Identifying Image-Derived Shoot and Root Traits with High Heritability and Repeatability

**Figure 4:**
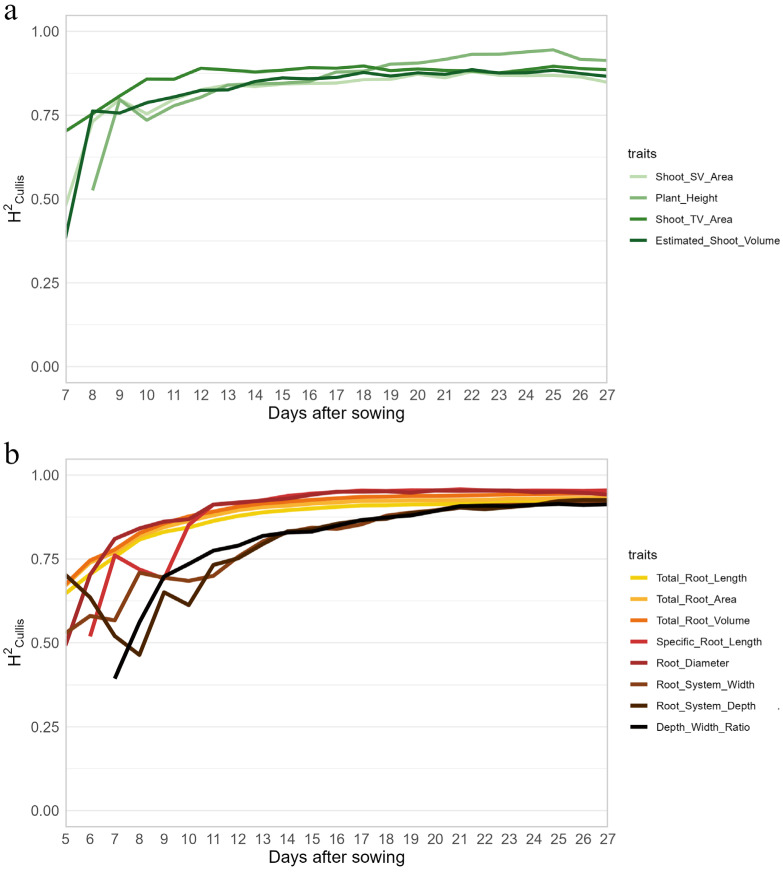
Heritability of image-derived shoot (**a**) and root traits (**b**) of the tested 65 maize inbred lines over time. Shoot_SV_area: side view area of the shoot, Shoot_TV_area: top view area of the shoot. Depth_Width_Ratio: the ratio of root system depth to width.

To assess the genetic contribution to the expression of image-derived shoot phenotypic traits and selected root traits over time, broad-sense heritabilities were calculated (Figure 4). After 12 DAS, most shoot and root traits reached heritability values of approximately 0.75 and thereafter they remained stable or slightly increased further. Because our study comprised only a single experiment/environment, repeatability was additionally calculated to assess the consistency of the measured traits across observations (Figure S1). Both shoot and root traits showed relatively low and unstable repeatability at the early developmental phase, but their repeatability gradually increased and stabilized after 13 DAS. Shoot biomass-related traits, such as ESV, reached repeatability values between 0.4 and 0.5, while PH reached values between 0.5 and 0.7. The highest repeatability values of root biomass-related traits were observed for TRL, TRA and TRV, with values of 0.63, 0.67, and 0.73, respectively. The SRL (specific root length) trait displayed high repeatability as well. Root system width (geometrical_x_dq95), root system depth (geometrical_y_p99_wo) and the ratio of depth to width (dep_wid_r) reached repeatability values higher than 0.5 after 19 DAS. We also extracted the variance components from the linear mixed models. Consistent with the repeatability results, both shoot and root traits showed approximately 50 % genotype contribution at 19 DAS, which remained stable thereafter (Table S2).

### 2.4. Growth Dynamics of Roots and Shoots Varied Among Lines and Across Growth Stages

**Figure 5:**
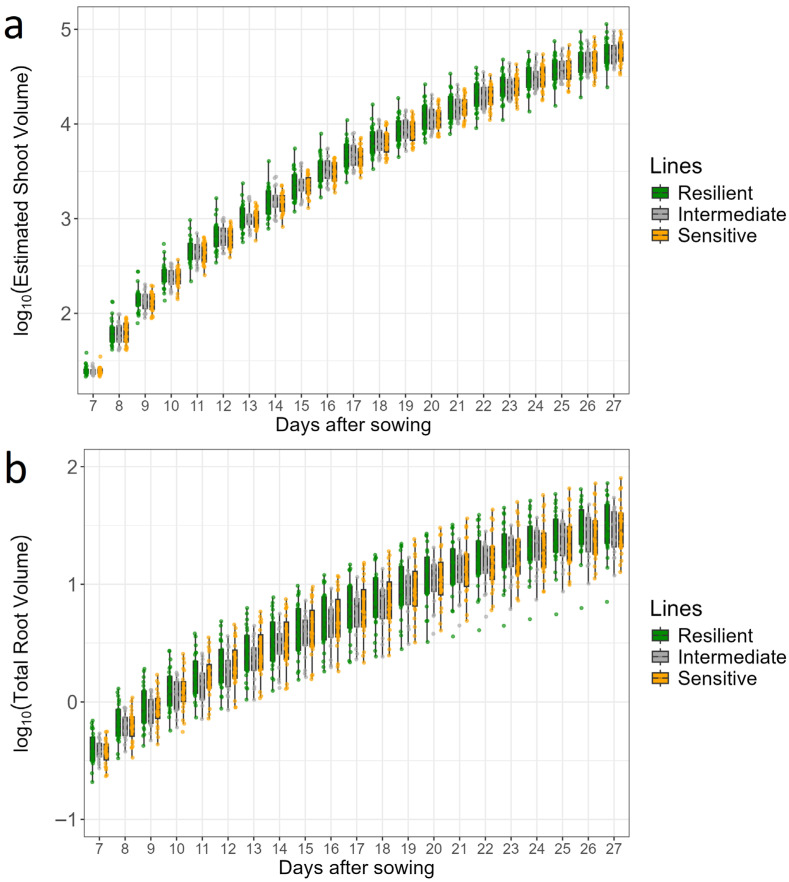
Estimated shoot volume (cm^3^) (**a**) and total root volume (cm^3^) (**b**) of the 65 tested maize lines over time. The lines were classified as resilient (25 lines), sensitive (23 lines), or intermediate (18 lines) in response to combined drought and heat stress according to [33]. The data shown are BLUPs for each line (dots), representing the predicted line values after adjusting for experimental effects.

During the cultivation, both TRL and TRV increased continuously (Figure S2), with notable genotypic variation for both traits among the tested lines. Lines EPD5, EPD125 and EPD2020-33 had the lowest TRL, as well as TRV at the latest stage (27 DAS). Lines Lo38, H101 and Lo1301 had the highest TRL, while H104, Lo1453 and NIL120 had the highest TRV. Some lines were consistently small (EPD5, EP125) or large (EP2018-08, Lo1301) in terms of TRV and kept their relative rank in comparison to the other lines over time. Dynamic shoot and root growth of the tested lines are presented in Figure 5. There was noticeable variation in both parameters across all days, which became more pronounced at later stages. No clear pattern could be observed that would allow the distinction of stress-resilient, sensitive or intermediate genotypes. The resilient line B107 and the sensitive lines EP68 and EPD5 had smaller shoot volumes than the other lines, while the resilient lines EP2018-08, Lo1453 and the sensitive line FP1 displayed overall large shoot volumes.

**Figure 6:**
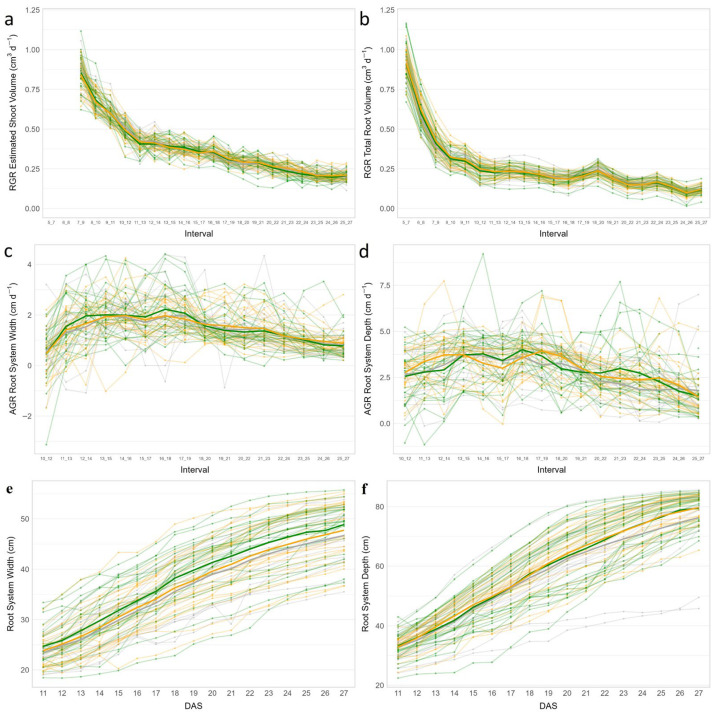
Shoot and root growth dynamics. (**a**) Relative growth rate (RGR) of estimated shoot volume and (**b**) RGR of total root volume. (**c**) Absolute growth rate (AGR) of root system width (geometrical_x_dq95) and (**d**) AGR of root system depth (geometrical_y_p99_wo) based on best linear unbiased prediction (BLUP) values of the 65 maize lines over time. Interval: growth rates were calculated over three-day intervals based on days after sowing (DAS). (**e**,**f**) Root system width and depth values measured over time (data obtained prior to 10 DAS were excluded because their very low repeatability rendered them unreliable). Transparent green, grey and yellow lines denote resilient, intermediate and sensitive genotypes, respectively. Mean values of these three groups were plotted as thick solid lines. Shoot phenotyping commenced with a two-day delay relative to root phenotyping.

Relative growth rates (RGRs) of both shoots and roots were calculated to assess growth dynamics and patterns among the tested lines over time, revealing dynamic patterns with the highest RGR observed during the early stages. In general, shoots demonstrated higher growth rates compared to roots (Figure 6a,b). RGR of roots differed among the lines and in different growth stages. The roots of EP68 grew very slowly, indicated by both TRL and TRV, at the early stage (5–7 DAS), but reached high TRL and TRV at 18–20 DAS and maintained these rates until 25–27 DAS. In contrast, the roots of NIL120 and Lo1301 grew substantially faster at 5–7 DAS, but these lines were among those with the slowest growth rates at 25–27 DAS. In addition, FP1 maintained relatively high root growth rates throughout early development (Figure 6b and Figure S3). To enable comparison of growth rates among lines independently of the overall sizes of the root systems and their changes over time, the absolute growth rates (AGRs) of root system width and root system depth for the 65 tested lines are presented in Figure 6c,d. Due to very large variation and low repeatability, data obtained prior to 10 DAS were not considered in the analysis. The AGRs of both root system width and depth increased during the early stage (10 to 18 DAS) and the maximum growth rate for both root system width and depth was reached at 16–18 DAS. Higher variation was observed in root system width compared to root system depth among the lines regarding both the measured values (Figure 6e,f) and the derived growth rates (Figure 6c,d). We selected 21 DAS for a comparative assessment of root properties, as at this advanced time point the root systems had developed characteristic differences, while the roots of most plants did not yet reach the margins of the rhizoboxes avoiding mechanical constraints to root growth. At this time point, the lines H104 and Lo1507 showed low root system width but substantial root system depth, indicating a deep rooting architecture. In contrast, the lines F252, EP2020-24 and TRL31 exhibited a large root system width but a limited root system depth, suggesting a wide or shallow RSA. Interestingly, the lines Lo863 and EP2020-31 displayed substantial root system width and depth, implying the development of an extensive root system that extends both vertically and horizontally, conferring the ability to exploit a particularly large soil volume (Table S3). Despite the observed RSA differences among individual lines, the three previously defined groups of stress-resilient, sensitive and intermediate lines did not show a clear pattern in the expression of their root traits, although there was a weak tendency that resilient lines had bigger root system width than the other groups (Figure 6e).

### 2.5. Multivariate Analysis Identified Clusters of Lines with Distinct RSA

**Figure 7:**
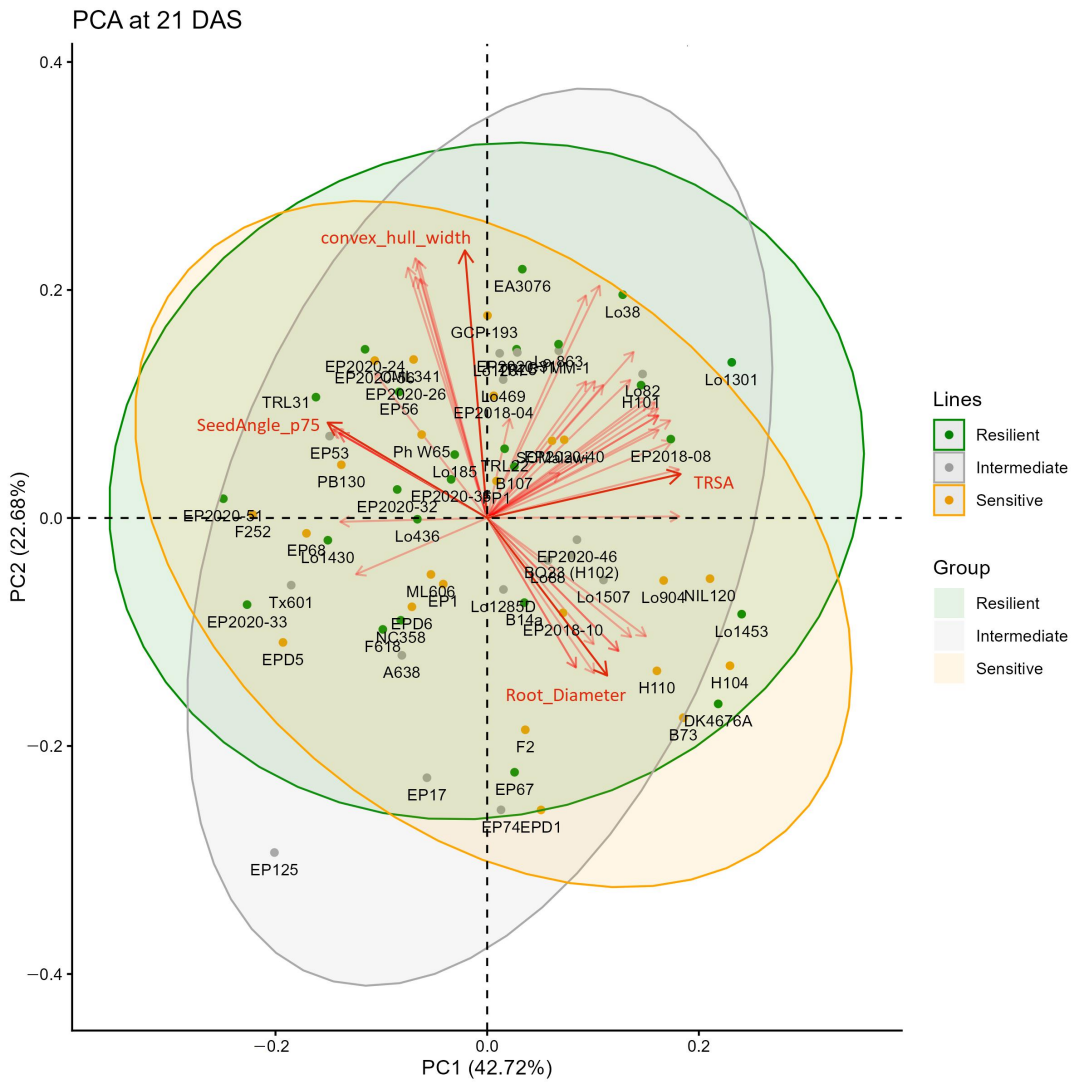
Principal component analysis (PCA) analysis of selected shoot (n = 4) and root traits (n = 40) of the 65 tested maize lines at 21 DAS (days after sowing) based on BLUPs. The lines were classified as resilient, sensitive and intermediate in response to combined drought and heat stress according to [33]. Red arrows indicated the trait loadings (traits) for PC1 and PC2, with the top four loadings labelled (dark red).

**Figure 8:**
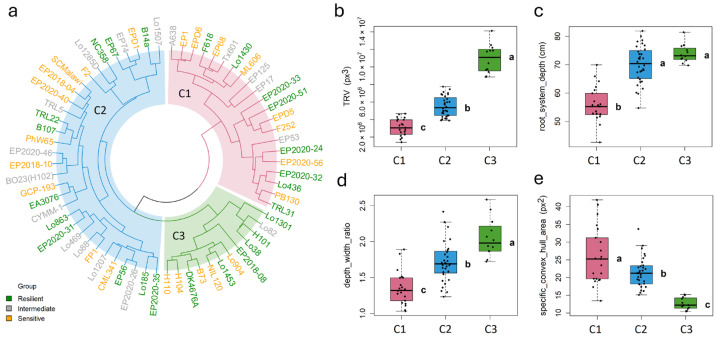
(**a**) Dendrogram of agglomerative hierarchical clustering of 65 maize lines on selected root (n = 40) and shoot (n = 4) traits at 21 DAS. Green, grey and yellow colours of the line names denote stress-resilient, intermediate and sensitive lines. The dendrogram is displayed in a circular layout. Branches were coloured to represent three major clusters (k = 3), with the red cluster (n = 21, C1) including lines with predominately shallow root system, the blue cluster (n = 32, C2) representing predominantly deep rooting lines, and the green cluster (n = 12, C3) corresponding to lines with a large root system, respectively. (**b**–**e**) The three groups are characterized by differences in total root volume (TRV), root system depth, depth_width_ratio, specific convex hull area, and several other traits. Letters shown beside the boxplots indicate statistically significant differences among clusters, determined by ANOVA followed by Tukey’s HSD test.

In order to evaluate the patterns among all image-derived phenotypic traits, a principal component analysis (PCA) was performed on the selected shoot and root traits measured at 21 DAS. To reduce effects of noise, traits with a genotypic variance contribution below 40% were excluded from the PCA. This particular day was selected because it corresponds to the period of high heritability/repeatability (19–27 DAS) and at this stage, the roots of most plants had not yet reached the margins of the rhizoboxes. PC1 and PC2 explain 42.72% and 22.68% of the variance, respectively. However, no clear separation of the three resilience groups was observed. Notably, line EP125 was positioned somewhat apart from the other lines (Figure 7). To identify natural groups and guide for further selection, agglomerative hierarchical clustering was performed using 44 selected image-derived shoot and root traits at 21 DAS (the same set of traits as used for the PCA, Figure 8a) and using PCA scores (Figure S4). The clustering patterns were largely consistent between the two approaches, indicating that dimensionality reduction did not substantially alter the overall grouping structure. The investigated lines were clustered into three mayor groups (Figure 8a). The first group, highlighted by green coloured branches in the dendrogram included lines with an overall large root system, indicated by high values of TRL, TRA, and TRV (Figure 8b). This group comprised the previously mentioned lines H104, Lo1453, Lo1301, and NIL120, among others. The second group, shown in red colour, were predominantly lines that displayed a shallow root system, such as EP125, EP17, EP2020-51, or F252. These lines were characterized by overall low values for root system depth (Figure 8c), while the third group (blue colour) contained deep rooting lines, including CYMM-1, EA3076 and EP2020-40. Although no clustering according to stress tolerance was observed, the grouping of lines based on RSA traits was also reflected in the first principal component (Figure 7), which captured the major variation associated with root system size and depth-related characteristics. Furthermore, the depth_width_ratio and specific convex hull area differed significantly between the three clusters (Figure 8d,e).

## 3. Discussion

Plant roots are essential multifunctional organs involved in the absorption and translocation of water and the uptake of nutrients. Variation in RSA provides an important means for plants to adapt to abiotic stresses, such as drought, and can be targeted in breeding programmes [31]. Integrating assessed root traits with genomic tools may offer new opportunities to develop high-performing, climate-smart crop varieties, but at the same time bears substantial challenges [38].

Sixty-five maize inbred lines, previously characterized for tolerance to combined drought and high temperature [33], were analyzed for their constitutive root growth and development in non-stress conditions to assess the degree of genetic variation and to address, as a first step in the investigation of stress-tolerance conferring root system architecture (RSA) features, the hypothesis of constitutive expression of root traits that are regarded as beneficial for upcoming adverse environmental conditions (pre-adaptation). Detailed RSA analysis was performed to assess relationships between root traits and stress tolerance in Mediterranean germplasm from diverse geographic regions, potentially shaped by different stress adaptations [39,40]. In the present study, automated high-throughput phenotyping revealed substantial variation in shoot and root traits, providing valuable data for an assessment of the relations of constitutive RSA properties with stress resistance properties. This is regarded as the first important step in the investigation of mechanisms providing stress resilience to be followed by studying acute responses of divergent genotypes to dynamic stress impacts (stress-induced plasticity). Nevertheless, the information on the sizes of the root systems and their deep or wide rooting properties, respectively, offer valuable selection criteria to select parental lines for breeding programmes directed to develop climate-resilient, locally adapted varieties.

At the time of harvest, there was approximately a 4-fold difference between the smallest and largest maize lines, based on manually measured shoot fresh and dry weight, and on image-derived estimated shoot volume (Figure 1 and Figure 5). RSA exhibited even greater variation, with the line displaying the highest total root volume (TRV) showing an 11-fold increase compared to the line with the lowest TRV. The repeatability of most tested traits varied during the cultivation/phenotyping period. A substantial number of traits exhibited lower repeatability at the early stages compared to later stages, which might have been caused by inhomogeneity in early seedling growth or technical limitations (e.g., data from small roots visible shortly after planting are more strongly affected by technical errors, such as erroneously segmented pixels, than the larger roots observed in the same area of the rhizoboxes). Among the root parameters extracted from the images, root biomass-related traits reached a stable repeatability greater than 0.5 at about 13 DAS. In contrast, other root traits, such as root system width, root system depth, exhibited repeatability values higher than 0.5 at 19 DAS. This suggests that root biomass is less affected by environmental factors than other root architecture-related traits. Liu et al. [41] reported that many root traits in field-grown maize plants had repeatability values around 0.6. However, these traits were measured at a much later growth stage (81 days after planting) compared to our study. As shown in Figure 4b, trait heritability (and repeatability; Figure S1) varied over time and tended to increase, becoming more stable at later developmental stages.

The root angle plays a key role in determining root distribution in the soil and is widely recognized as an important trait linked to drought tolerance [42,43]. Genetic variation in root angle has been reported in barley, sorghum, oilseed rape, as well as maize [11,21,44,45]. Under our experimental conditions, a near 2-fold difference was observed between the lines with the widest (137°) and narrowest (78°) root angles (Figure 2). Extracting root angle data from images of soil-grown roots is often challenging due to root occlusion within the soil and the limitations of two-dimensional imaging. Rhizotron images capture only 2D growth, which may not fully reflect natural root development. For traits like root angle, 3D models may provide more accurate and biologically representative measurements. However, their application in high-throughput rhizotron systems is currently limited by technical constrains and the inherently planar growth conditions imposed by the system. Consequently, rhizotron-based root angle measurements remain largely dependent on manual analysis. In this study, the ratio of root system depth to root system width appeared to serve as a valuable indicator of root angle, given its high correlation with manually measured root angles r = −0.58 ***). Besides that, root system width might also be another promising indicator for root angle, given its high correlation. However, additional research is required to assess the accuracy of image-derived root angle measurements in comparison to the true root angle, ideally derived from 3D RSA data.

The automated high-throughput root phenotyping (Rhizotron) facility enabled us to assess the dynamics of root growth of divergent genotypes under controlled environmental conditions. The change rates of maize root traits (TRL, TRV, root system depth and root system width) were higher and showed greater variation among the lines during the early stages. Some lines exhibited already longer roots or larger root volumes at the beginning of the imaging (5 DAS) and maintained high root growth rates, while others displayed a decline in growth rate and failed to develop a large root system (Figure 6). This suggests that maternal effects and seedling vigour might partly contribute to root system development, particularly during very early stages, but the main root architecture is primarily genetically controlled as reported by many previous studies [8,10,15,27,37]. Notably, the studied lines displayed distinct RSA with respect to root system width and depth development. Certain lines developed their roots more in the vertical direction, showing a preference for deep rooting (e.g., H104, Lo1507), whereas others tended to form wider and shallower root systems (e.g., F252, EP2020-24, TRL31). As previously reported [7,37], a steeper root system enables access to mobile water and nitrogen from deeper soil layers, whereas shallow root systems facilitate the capture of immobile phosphorus from the topsoil. Moreover, two lines (Lo863 and EP2020-31) were capable of developing both a high root system depth and width. According to our previous work, both lines were classified as resilient to combined drought and heat stress [33]. However, it should be considered that lines capable of developing larger root systems under non-stress conditions, where resources are abundant, may not maintain an advantage under stress conditions with limited resources, as they require greater energy expenditure [46,47]. Therefore, the performance of these lines under abiotic stress conditions and their abilities to respond with stress-induced expression of favourable traits (stress-induced plasticity) need to be further studied. Nevertheless, the information of the varying constitutive RSA properties of the characterized lines is a very valuable input for the selection of parental lines for breeding devoted to the generation of locally adapted stress-resilient varieties. In regions with intensive agricultural management including irrigation, shallow root systems that efficiently capture the water supplied at the soil surface may enhance performance in combination with other resilience adaptations, whereas in purely rain-fed environments, deep root systems may offer superior resource acquisition.

Among the 65 lines tested, 25 were classified as resilient and 23 as sensitive to combined drought and heat stress, based on our former work [33]. Since previous studies reported that shoot and root biomass are positively correlated under either control or stress conditions [11,16], it was hypothesized that stress-resilient genotypes may already exhibit larger root systems under non-stress conditions compared to sensitive genotypes. However, our study did not yield evidence to support this hypothesis, instead suggesting that multiple strategies including capacities of stress-induced dynamic developmental and physiological responses are involved in stress adaptation. Although there was a strong positive correlation between ESV and TRV (Figure 3), the rankings of lines for these two traits were not identical. Moreover, PCA suggested that the three groups of resilient, intermediate and sensitive lines cannot be distinguished by the extracted root and shoot traits under our experimental conditions (Figure 7). Nevertheless, there was a significant negative correlation (r = −0.29 *) between the stress resistance index and the root diameter. This suggests that resilient lines tend to produce thinner roots or exhibit increased lateral branching. Thick roots contribute to anchorage and help penetrate hard soils, whereas a high density of thin or lateral roots may enhance the plant’s ability to explore the soil and acquire both mobile (including water) and immobile resources [48]. According to a recent report [34], large root systems do not consistently lead to higher biomass, especially under drought conditions in maize. This indicates that resilient maize lines employed different strategies to adapt to combined drought and/or heat stress, and not all of them rely on the root system. It has been reported that some drought resilient maize lines were able to keep high photosynthesis and low transpiration rates or increase osmotic potential in leaves [5,49]. Furthermore, plant adaptation to abiotic stress involves coordinated morphological and physiological responses in shoots and roots, as well as shoot–root signalling. Plants sense environmental challenges and adjust growth accordingly, with responses regulated by complex multi-level molecular mechanisms including sensing, signal transduction, transcription, and metabolic adjustments [50,51]. These findings are consistent with our results, which indicate that resilience is multifactorial and not solely determined by root system size or architecture properties already expressed in non-stress conditions (pre-adaptation).

On the other hand, in our previous study, stress adaptability was tested in small pots which limited root growth and consequently affected the contribution of root traits to stress resistance. Root systems are highly plastic and responsive to their environment [24,43]. This phenotypic plasticity is an important phenomenon to enable plants to dynamically adjust to spatiotemporal changes [24]. A limitation of the present study is that we did not investigate adaptive plasticity. This may further explain why the resilient and sensitive lines could not be distinguished. The next step in a comprehensive investigation of the stress resilience mechanisms of selected divergent lines therefore need to include the examination of adaptive stress-induced plasticity, such as alterations in RSA under stress conditions. We consider it crucial to elucidate the role of root adaptive plasticity in stress resilience as part of our upcoming work.

It is important to note that most components of RSA are influenced by both genetic and environmental factors, as well as their interaction. For instance, higher expression of the DEEP ROOTING (DRO1) gene discovered in rice [42,52] has been reported to promote a steeper root angle, which proves advantageous under drought conditions. In contrast, the loss of its homologue, qSOR1, results in a shallower root angle that confers a higher tolerance to saline soils. This example illustrates that a better understanding of the genetic regulation of root angle and other root-related traits can have a crucial impact for selective breeding of stress-adapted plants. The root angle is influenced by multiple factors, and its regulation may also be affected in a root type-specific manner [37]. Therefore, genetic variation in root angle under particular environmental stress conditions should also be taken into account. Among the lines with the top ten narrowest root angles, there were five resilient, four sensitive, and one intermediate, while there were four resilient, four sensitive, and two intermediate lines among the top ten lines with the widest root angle. This suggests that when selecting stress-resilient maize lines, multiple traits should be considered, and it may be advantageous to include both shoot and root traits.

Despite being conducted under non-stress conditions, the in-depth analysis of constitutive root growth patterns in Mediterranean maize germplasm highlights RSA diversity that is valuable for breeding (Figure 8). The stable root traits identified in this study can be leveraged to detect potential quantitative trait loci in more dedicated mapping populations or to train genomic prediction models using larger panels. These results provide a basis for selecting root systems that may contribute to above-ground biomass accumulation and maintain consistent performance across environments, offering potential utility for future breeding strategies. Additionally, these traits could guide future research aimed at developing maize varieties with potentially enhanced resilience to drought and heat in stress-prone agroecologies through the targeted introgression of beneficial root traits into elite germplasm. Lines classified as resilient, based on their high stress adaptability in shoot traits, represent strong candidates for deployment across diverse agroecological conditions. In well-managed (irrigated) environments, lines combining an optimal RSA, such as wide root angles and rapid lateral root system expansion, offer greater potential for selection and breeding for stress resilience. In contrast, in rain-fed or low-irrigation environments, the integration of narrower root angles and rapid root system depth development would be more advantageous. In this context, our high-throughput phenotyping system installed in a climate-controlled environment will be instrumental for future studies.

## 4. Materials and Methods

### 4.1. Plant Material and Phenotyping

A total of 65 Mediterranean maize inbred lines were selected based on our previous experiments and pedigree information of the inbred lines detailed in Shi et al. [33]. The lines were classified as either stress-resilient, intermediate, or stress-sensitive based on stress indices calculated from data generated under both non-stress and stress conditions (combined drought and heat) in a controlled environment (Table S1).

Root system architecture (RSA) of the lines was evaluated by using the automated high-throughput root phenotyping platform (rhizotron system) installed inside the climate-controlled environment of the IPK-PhenoSphere at the Leibniz Institute of Plant Genetics and Crop Plant Research (IPK), Gatersleben, Germany. Maize seeds were germinated on moist filter paper in the dark for three days before being transplanted into rhizotrons. Each line was replicated four times, with one plant per rhizotron (about 90 × 60 × 4 cm, height × width × thickness). Each rhizotron was filled with 10 kg of substrate (Potgrond, Klasmann, Germany). Plants were grown at 25 °C day/20 °C night (16/8 h day/night), with 70% relative air humidity and automated watering, maintaining 40% field capacity.

During the cultivation in the rhizotron system, automatic watering was performed daily based on target weight. Meanwhile, shoot imaging from top and side were captured using RGB cameras (Manta G-1236, Stadtroda, Germany), and root images were obtained using a monochrome camera (Prosilica GT 6600, Stadtroda, Germany). All images were acquired in an imaging tower that moves during the watering and imaging processes. After 24 days of growth in the system (27 days after sowing; DAS), shoots were cut and sampled for the analysis of fresh weight (FW) and dry weight (DW; drying in an oven at 70 °C for one week).

### 4.2. Automated Image Analysis and Image-Derived Traits

The obtained shoot images were analyzed using DeepShoot software https://ag-ba.ipk-gatersleben.de/ds.html (accessed on 15 March 2026) [35]. Root images were analyzed by fully automated Root Image Analysis software (faRIA) https://dx.doi.org/10.1038/s41598-021-95480-y (accessed on 15 March 2026) [36]. Four shoot traits, PH (plant height), Shoot_SV_area (side view area of the shoot), Shoot_TV_area (top view area of the shoot), Shoot_volume (estimated shoot volume) and 48 root traits (detailed root traits definition can be found in [47]) were extracted for each day. Some new parameters were updated from faRIA software. The parameters with the name tag ‘wo’ and ‘dq’ indicate ‘without outliers’ (after outlier correction) and ‘differential quantiles’, respectively (Supplementary Information File S1). One additional root trait, the ratio of depth to width (dep_wid_r) was calculated by dividing geometrical_x_dq95 by geometrical_y_p99_wo. Relative growth rate (RGR) of shoots and roots were calculated based on image-extracted shoot volume (ESV) or total root volume (TRV) in overlapping 3-day intervals according to the following equation:

RGR = (ln (EV2)-ln (EV1))/t2-t1 (EV: ESV or TRV, t: time, day). Absolute growth rate (AGR) of root system width and depth were calculated by the equation: AGR = (width2 or depth2-width1 or depth1)/(t2-t1). Here t denotes days after sowing time.

The root angle (RA_m) was measured manually from images at the last imaging time point at 27 DAS using Image J (https://imagej.net/ij/ (accessed on 15 March 2026)) as described by Jia et al. [44]. The total root number (TRN), which included primary root, seminal roots and crown roots, was determined manually.

### 4.3. Statistical Analysis

A linear mixed model was used to assess best linear unbiased predictors (BLUPs) for all image-derived phenotypic traits using the lme4 R package [53]. The model is as follows: y = µ + Gen + Lane + Rep + ε, whereby y represents the phenotypic traits, Gen the genotype effect, Lane corresponds to the lanes in the rhizotron system (the position effect), Rep denotes the replicate effect and ε the residual error. To estimate variance components, all factors, including genotype, were modelled as random effects, since the genotypes were considered as a sample from a broader population rather than fixed treatment levels. Repeatability was calculated as follows: repeatability = σ_α_2/(σ_α_^2^ + σ_ɛ_^2^) according to Nakagawa and Schielzeth [54]. Broad-sense heritability was calculated from the same linear mixed model according to Cullis and Schmidt [55,56], $H_{Cullis}^{2}=1-\frac{{\overset{-}{\upsilon }}_{\Delta ..}^{BLUP}}{2\sigma_{g}^{2}}$, ${\overset{-}{\upsilon }}_{\Delta ..}^{BLUP}$ is the mean variance of a difference of two BLUPs for the genotypic effect and $\sigma_{g}^{2}$ denotes the genotypic variance.

Differences in shoot FW and DW between lines were assessed using a one-way ANOVA at alpha = 0.05. Principal component analysis and agglomerative hierarchical clustering of maize genotypes were performed using 44 standardized root and shoot traits (BLUPs) measured at 21 DAS. Prior to analysis, selected non-informative variables were removed. For clustering, trait values were z-score standardized, and Euclidean distances among genotypes calculated. The resulting dendrogram was partitioned into three clusters (k = 3). For visualization, genotype labels were coloured according to previously determined stress response classification (green = resilient, grey = intermediate, orange = sensitive) [33], and the dendrogram was displayed in a circular layout using the circlize R package. Differences between clusters for individual traits were assessed using ANOVA followed by Tukey’s HSD test. The data visualization for phenotyping data, statistical analysis and cluster analysis were all performed using R software version 4.4.2 [57].

## 5. Conclusions

In this study we characterized the constitutive root system architecture and shoot biomass production of 65 maize lines from Mediterranean regions during early vegetative development. Significant genotypic variation was observed not only in root biomass-related traits (TRL, TRA, TRV) and root architectural traits (root angle, root system width, root system depth), but also in the relative growth rates. Stress-resilient and stress-sensitive lines could not be distinguished based on RSA traits expressed under non-stress conditions, indicating that stress tolerance is not determined by a predefined uniform set of traits, but rather by diverse strategies for coping with drought and heat stress. These findings provide a solid foundation for further investigations including dynamic responses to stress conditions and can assist breeders in identifying promising parental lines (genotypes) for new breeding programmes devoted to the development of varieties with improved heat and drought tolerance, greater root biomass, and enhanced nutrient use efficiency. Lines exhibiting distinct RSA were characterized, highlighting their significant potential for developing varieties adapted to local environments and through enhanced expression of favourable root-related traits.

## Data Availability

The original contributions presented in this study are included in the article/Supplementary Material. Further inquiries can be directed to the corresponding author.

## Supplementary Materials

The following supporting information can be downloaded at: https://www.mdpi.com/article/10.3390/plants15060935/s1, Figure S1: Repeatability of image-derived shoot (a) and root traits (b) of the tested 65 maize inbred lines over time. Shoot_SV_area: side view area of the shoot, Shoot_TV_area: top view area of the shoot. Depth_Width_Ratio: the ratio of root system depth to width; Figure S2: (a) Estimated total root length (TRL) and (b) total root volume (TRV) derived from the images of the tested 65 inbred maize lines over time. Data are shown as BLUPs for each line. DAS: days after sowing; Figure S3. Relative growth rate (RGR) of TRL (total root length) of the tested 65 maize inbred lines over time (represents as three days interval); Figure S4. Dendrogram of agglomerative hierarchical clustering of 65 maize lines on PCA scores (PC1, PC2, PC3 and PC4) performed at 21 DAS. Green, grey and yellow colours of the line names denote stress-resilient, intermediate and sensitive lines. The dendrogram is displayed in a circular layout; Table S1. Overview of the 65 maize inbred lines used in this study; Table S2. Genotypic contribution to phenotypic variation in image-derived shoot and root traits over time; Table S3. The 15 maize inbred lines exhibited the lowest and highest relative growth rates (RGRs, px/day) in root system depth and root system width among the 65 lines tested; Supplementary Information File S1: Description of root system architecture (RSA) traits estimated using faRIA software.
