# Supplementary material for: Diversity of Root System Architecture in Mediterranean Maize Inbred Lines Provides New Breeding Opportunities to Improve Stress Resilience and Resource Efficiency
Source: Plants (Basel). 2026 Mar 18;15(6):935. doi: 10.3390/plants15060935 (PMC13030489; doi:10.3390/plants15060935)
Supplement: Supplementary file 1 [file plants-15-00935-s001.zip › Supplementary figures.pdf]

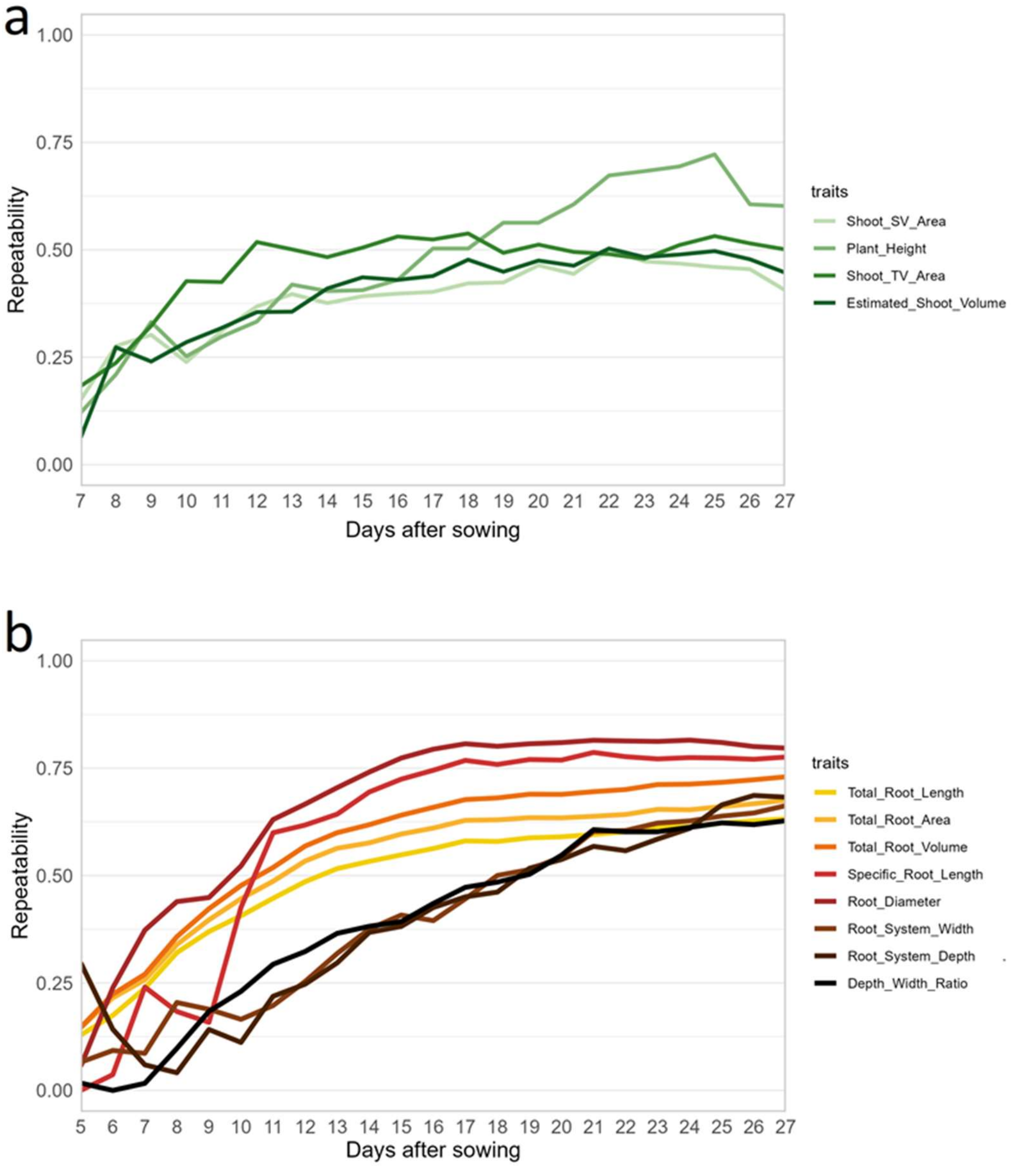

Figure S1. Repeatability of image-derived shoot (a) and root traits (b) of the tested 65 maize inbred lines over time. Shoot_SV_area: side view area of the shoot, Shoot_TV_area: top view area of the shoot. Depth_Width_Ratio: the ratio of root system depth to width.

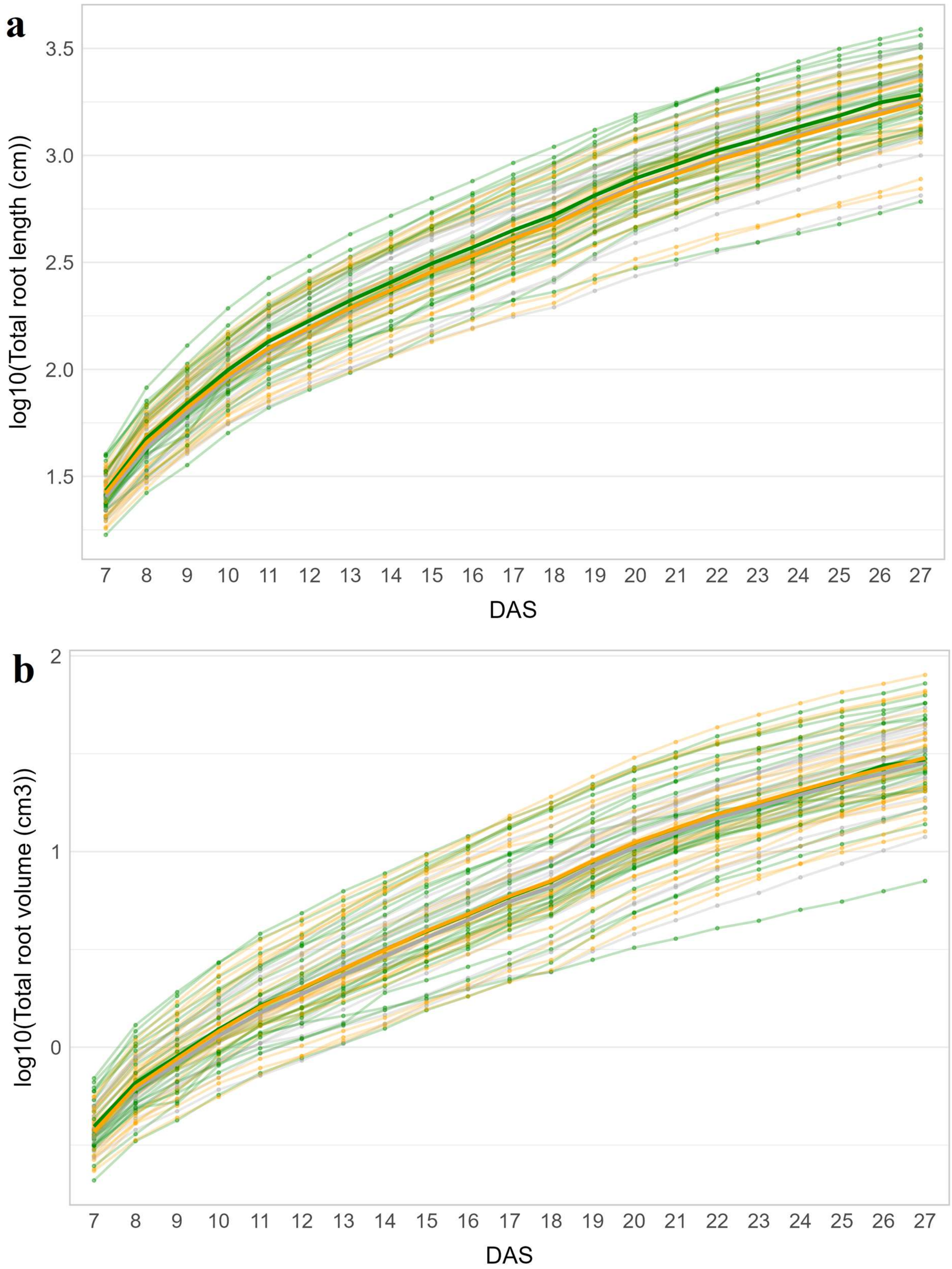

Figure S2. a) Estimated total root length (TRL) and b) total root volume (TRV) derived from the images of the tested 65 inbred maize lines over time. Data are shown as BLUPs for each line. DAS:

days after sowing. Green, grey and yellow lines denote resilient, intermediate and sensitive genotypes using transparent lines, respectively. Mean values of these three groups were plotted using solid lines.

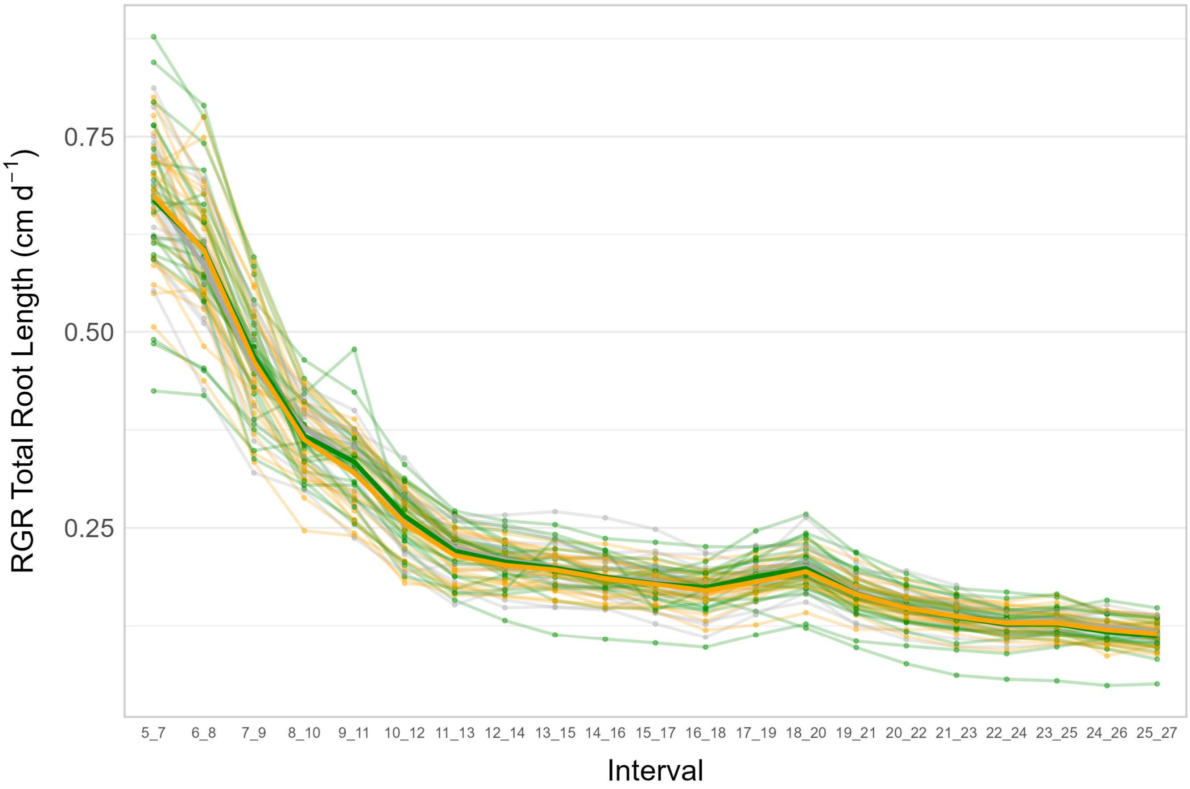

Figure S3. Relative growth rate (RGR) of TRL (total root length) based on best linear unbiased prediction (BLUP) values of the 65 maize lines over time. Intervals were calculated over three days based on days after sowing (DAS). Green, grey and yellow lines denote resilient, intermediate and sensitive genotypes using transparent lines, respectively. Mean values of these three groups were plotted using solid lines.

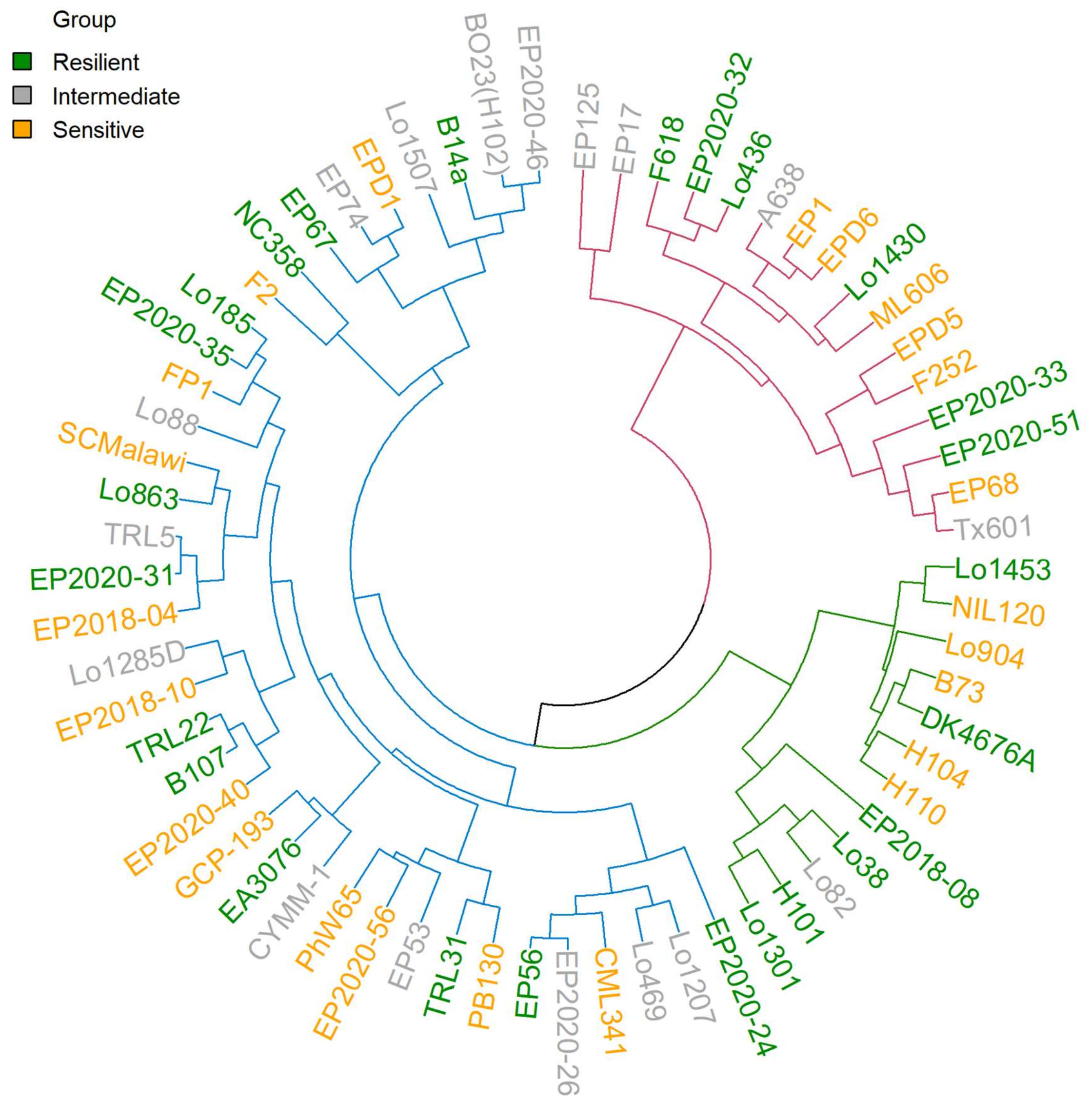

Figure S4. Dendrogram of agglomerative hierarchical clustering of 65 maize lines on PCA scores (PC1, PC2, PC3 and PC4) performed at 21 DAS. Green, grey and yellow colours of the line names denote stress resilient, intermediate and sensitive lines. The dendrogram is displayed in a circular layout.
