# Supplementary material for: Diversity of Root System Architecture in Mediterranean Maize Inbred Lines Provides New Breeding Opportunities to Improve Stress Resilience and Resource Efficiency
Source: Plants (Basel). 2026 Mar 18;15(6):935. doi: 10.3390/plants15060935 (PMC13030489; doi:10.3390/plants15060935)
Supplement: Supplementary file 1 [file plants-15-00935-s001.zip › Supplementary Information File S1.pdf]

Description of Root System Architecture (RSA) Traits Estimated Using *faRIA* Software

Table 1: Basic Root Traits

Trait	Description
Area	Total number of root pixels in the image
Total Length	Number of skeleton pixels representing the full root system
Total Volume (V)	Sum of volumes of all root segments (assumed tubular, based on average radius)
Total Surface Area (SA)	Sum of surface areas of root segments (tubular shape using average radius)
Number of Regions	Number of disconnected root segments in the image
Number of Branching Points	Total number of branches in the root skeleton
Number of End Points	Total number of terminal points in the root skeleton
Specific Root Length	Ratio of total root length to total volume

Table 2: Geometrical Traits (X and Y directions)

Trait	Description
X_mean, Y_mean	Mean position of root pixels
X_median, Y_median	Median position of root pixels
X_std, Y_std	Standard deviation of root pixel distribution
X_skew, Y_skew	Skewness (asymmetry) of root pixel distribution
X_kurt, Y_kurt	Kurtosis (peakedness) of root pixel distribution
X_pNN, Y_pNN	NN percentiles (e.g., p25, p99) of pixel positions in horizontal and vertical axes
X_dqNN, Y_dqNN	NN central differential quantile (e.g., dq25, dq99) of pixel positions in horizontal and vertical axes
X/Y_bootstrap_mean, X/Y_bootstrap_stdev	Bootstrapped mean and standard deviation of root pixel positions

Note: Traits marked with `_wo` indicate values calculated after denoising segmented images.

Table 3: Root Diameter Traits

Trait	Description
Diameter mean / median / std	Mean, median, and standard deviation of root diameter
Diameter skew / kurt	Skewness and kurtosis of root diameter
Diameter pNN	NN Percentile (e.g., p25, p99) values of root diameter
Diameter bootstrap_mean / median	Bootstrapped mean and median root diameter

Table 4: Root Orientation Traits

Trait	Description
Orientation mean / median / std	Mean, median, and standard deviation of root orientation angles
Orientation skew / kurt	Skewness and kurtosis of orientation angles
Orientation pNN	NN percentiles (e.g., p25, p99) of orientation angles
Orientation bootstrap_mean / median	Bootstrapped statistics for orientation angles

Table 5: Seed Angle Traits (relative to seed point)

Trait	Description
SeedAngle mean / median	Mean and median orientation relative to the seed point
SeedAngle skew / kurt	Skewness and kurtosis of angles relative to the seed point
SeedAngle pNN	NN percentiles (e.g., p25, p99) of orientation relative to the seed point
SeedAngle bootstrap_mean / stdev	Bootstrapped mean and standard deviation of seed-relative orientation
Seed Opening Angle	Estimated RSA opening angle from seed point, based on root pixel distribution

Table 6: Convex Hull Traits

Trait	Description
ConvexHull area	Area of the convex hull enclosing the root system
ConvexHull width / height	Width and height of the convex hull
ConvexHull specific_area	Ratio of convex hull area to actual root system area
